# Cohort study of Anticoagulation Self-Monitoring (CASM): a prospective study of its effectiveness in the community

**DOI:** 10.3399/bjgp15X685633

**Published:** 2015-06-29

**Authors:** Alison Ward, Alice Tompson, David Fitzmaurice, Stephen Sutton, Rafael Perera, Carl Heneghan

**Affiliations:** Nuffield Department of Primary Care Health Sciences, University of Oxford, Oxford.; Nuffield Department of Primary Care Health Sciences, University of Oxford, Oxford.; University of Birmingham, Edgbaston.; Institute of Public Health, University of Cambridge, Cambridge.; Nuffield Department of Primary Care Health Sciences, University of Oxford, Oxford.; Nuffield Department of Primary Care Health Sciences, University of Oxford, Oxford.

**Keywords:** anticoagulants, primary care, monitoring, self-management, self-monitoring

## Abstract

**Background:**

Trials show that oral anticoagulation therapy (OAT) substantially reduces thromboembolic events without an increase in major haemorrhagic events, but it is not known whether these results translate into routine practice.

**Aim:**

To estimate the current levels of control and adverse events in patients self-monitoring OAT, explore the factors that predict success, and determine whether the level of side effects reported from randomised controlled trials are translated to a non-selected population.

**Design and setting:**

Prospective cohort study in the UK.

**Method:**

Participants were aged ≥18 years and registered with a GP. Main outcomes were the proportion of participants, over 12 months, who were still self-monitoring, had not experienced adverse events, and had achieved >80% of time in therapeutic range (TTR).

**Results:**

In total, 296 participants were recruited; their median age was 61 years and 55.1% were male. Participants were predominately professional or held a university qualification (82.7%). At 12 months, 267 (90.2%) were still self-monitoring. Mean TTR was 75.3% (standard deviation 16.9).Six serious and two minor adverse events were reported by GPs. Only 45.9% of participants received any in-person training at the outset. Increased age (*P* = 0.027), general wellbeing (EQ-5D visual score, *P* = 0.020), and lower target international normalised range (INR, *P* = 0.032) were all associated with high (>80% TTR) levels of control.

**Conclusion:**

The findings show that, even with little training, people on OAT can successfully self-monitor, and even self-manage, their INR. TTR was shown to improve with age. However, widespread use of self-monitoring of INR may be limited by the initial costs, as well as a lack of training and support at the outset.

## INTRODUCTION

Oral anticoagulation therapy (OAT) substantially reduces thromboembolic events with no increase in major haemorrhagic events.^1^–^5^ However, uptake is still affected by the need to maintain the international normalised ratio (INR) in a narrow range, which requires frequent testing and dose adjustment. Reliable and accurate point-of-care devices enable patients to test their INR in their own setting. They can either self-monitor their INR and have their oral anticoagulation dosage managed by a healthcare provider or they can self-manage and adjust their own dosage.

Patients have been found to vary considerably in their ability to self-monitor and self-manage. Results of randomised controlled trials (RCTs) show that only 38% (range 12–59%) of those initially identified as potentially eligible for self-monitoring of OAT actually participated in the trials; in addition, only 78% assigned to the intervention continued self-monitoring until the end of the trial.^6^

Current guidelines recommend self-monitoring or self-management for patients who have long-term indications, a recognised target INR, and have received appropriate training by a health professional.^2^,^7^ The outcomes of the trials for those that participate fully have been positive for both self-monitoring and self-managing OAT,^3^ but it is not known whether these results translate into routine practice. This study aimed to:

- prospectively estimate the current levels of control and adverse events in patients self-monitoring OAT in the UK;
- explore the factors that predict successful self-monitoring of INR;
- ascertain whether the success of INR self-monitoring and level of side effects reported in RCTs of self-monitoring INR are translated to a non-selected population; and
- estimate the adequacy of existing training and quality-assurance arrangements.

## METHOD

### Study population and recruitment

The study population consisted of all people who purchased an INR self-monitoring device from the main distributor in the UK. During the study period, this distributor had 99.9% of the market share.

All people aged ≥18 years, who were able to give informed consent, comprehend English sufficiently well to be interviewed, and registered with a UK GP were eligible to take part. Recruitment lasted from February 2009 until August 2011. Originally, only those who were new to self-monitoring — defined as self-monitoring for ≤3 months — were recruited. However, due to low numbers, recruitment was broadened to include people who had been self-monitoring for some time and were purchasing replacement devices.

**How this fits in**

Oral anticoagulation therapy (OAT) greatly reduces thromboembolic events without increasing major haemorrhagic events. The outcomes of a number of trials have been positive for both self-monitoring and self-managing oral anticoagulation, but it is not known whether these results translate into routine practice. This study shows that, even without much training, individuals on OAT can self-monitor, and even self-manage, their international normalised ratio (INR) with positive results outside of trial settings. Such patients could be offered self-monitoring and self-management of their OAT with suitable healthcare support. However, it should be noted that participants in this study were highly educated so caution should be taken with regard to generalising the findings to the broader population.

### Sample size

As the primary aim of the study was to obtain an adequate estimate of the rate of successful self-monitoring, the sample size calculation was based on a certain precision around this estimate (equal to 1.96 standard errors, or one side of a 95% confidence interval [CI]). The worse-case scenario in terms of precision, given a rate of 50% (maximum variance) and a 20% attrition, would require 300 participants to achieve a precision of ±6% around the estimate (for example, 95% CI = 44 to 56). A rate of 35% (or 65%) based on the same sample would give a precision of ±5.4%. Rates of <35% (or >65%) would give better precision levels. This sample also allows for the identification of predictive variables with odds of greater than approximately 2.5 — for example, an odds ratio [OR] of 2.3 for an event of 70% in the successful group, versus 50% in the unsuccessful group — with similar confidence.

### Baseline telephone interviews and questionnaires

Consenting participants were sent a postal questionnaire and telephoned to complete a baseline interview. Details requested covered:

- basic demographics;
- anticoagulation history;
- self-monitoring training;
- support;
- adverse events;
- quality assurance;
- comorbidity;
- medications;
- attitudes towards self-monitoring of INR;
- confidence about self-monitoring INR; and
- psychosocial measures.

The psychosocial measures comprised the EQ-5D,^8^ Conscientiousness scale,^9^ Morisky Adherence to Medication Scale,^10^ National Institute of Health and Care Excellence (NICE) Depression Screening Tool,^11^ State-Trait Anxiety Inventory,^12^ Hospital Anxiety and Depression Scale,^13^ Brief Illness Perception Questionnaire,^14^ an adapted Treatment Self-Regulation Questionnaire including perceived competence/self-efficacy scale and Health Care Climate Questionnaire,^15^ (based on the theory of self-determination)^16^ and the Theory of Planned Behaviour Questionnaire.^17^

### Interview schedule

Face-to-face interviews were conducted with a small number of participants. Interviews were semi-structured with open-ended questions to elicit new information; they were recorded using digital audio equipment and transcribed. Questions explored the:

- experiences of self-monitoring, focusing on the barriers and facilitators;
- reasons for not starting, or not continuing, self-monitoring;
- training received; and
- quality assurance.

### Follow-up data collection

Participants were contacted by telephone at 3, 6, and 12 months to ascertain whether they were still self-monitoring; a brief questionnaire explored their experiences of doing so. At 12 months, participants were also sent a validated questionnaire to assess their oral anticoagulation knowledge.^18^ During follow-up, they recorded their INR data in a log book and posted it to the study coordinator every 3 months. Clinical data for participants for the 12-month study period were ascertained from GP medical records.

Two GPs independently classified the reported adverse events using the bleeding severity index;^19^ differences were resolved by discussion.

### Data analysis

Statistical analyses were performed using SPSS (version 20). Comparisons of descriptive data were conducted using χ^2^ tests for categorical variables and binary logistic regression for continuous variables. The proportion of participants continuing to self-monitor over time was ascertained from the interview data, while the proportion of time in therapeutic range (TTR) was calculated using the INR data. Percentage TTR (%TTR) was calculated using Rosendaal *et al*’s method^20^ in Stata (version 11).

Successful self-monitoring was defined as the proportion of participants:

- continuing to self-monitor for 12 months;
- not experiencing adverse events over the 12 months; and
- achieving >80%TTR.

In the calculation of TTR, the participant’s reported target range was used for analyses; participant-reported INR tests conducted by health professionals were excluded. Duplicate tests carried out on the same day were averaged. The start date was taken as the date of the baseline interview; the end date was 365 days later. Participant INR data was excluded if the submitted test results spanned <90 days in total during the study period. TTR was not interpolated for the missing periods. Data with a reported gap between tests of >12 weeks was treated as intermittent.

Forward stepwise logistic regression was used to establish potential predictors of successful self-monitoring. Only baseline characteristics were included in the model; the addition of whether a participant had experienced a surgical procedure during the 12 months was excepted as this was likely to reduce TTR. Age, sex, condition requiring oral anticoagulation, duration of self-monitoring, surgical procedure during follow-up, and previous oral anticoagulation complications were all entered into a model before the addition of baseline variables with a univariate OR *P*-value of <0.10.

## RESULTS

**Figure 1.**
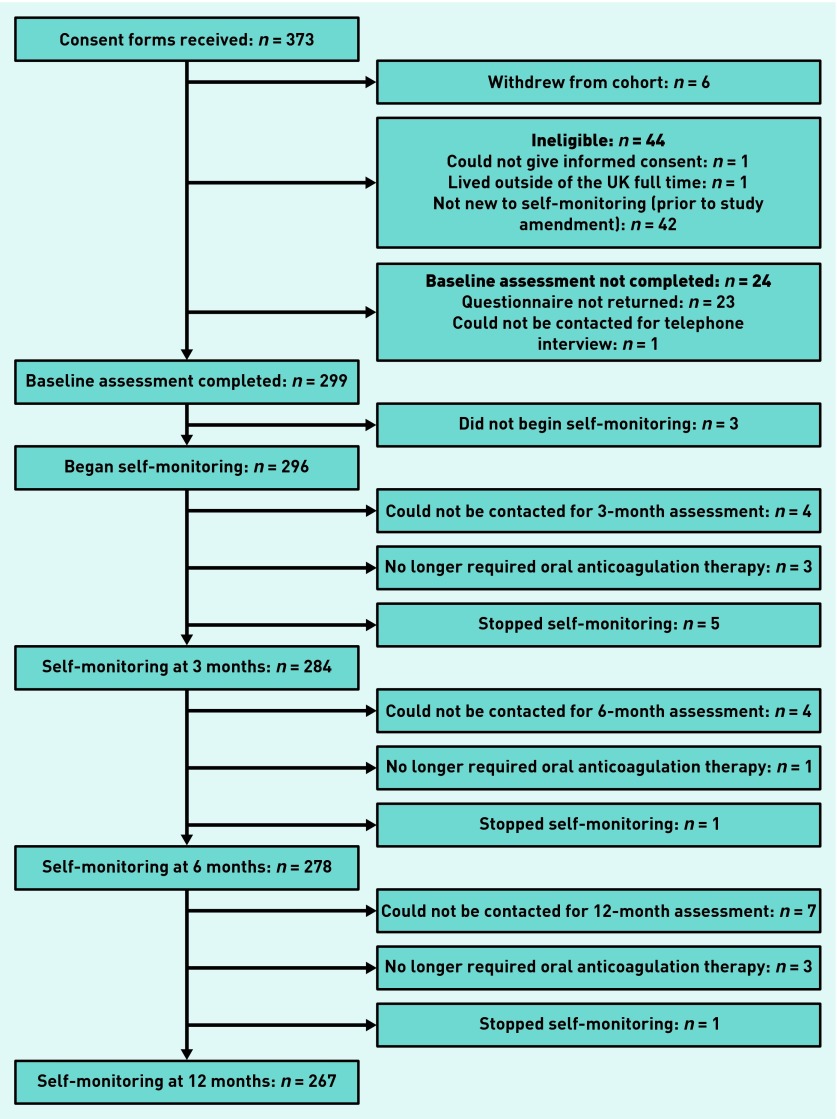
***Flow chart of recruitment process.***

Of the individuals approached to participate in the study, 299 were eligible, completed the baselines questionnaires, and were recruited. Of these, three participants did not go on to begin self-monitoring (two due to lack of support from their healthcare provider and one for unknown reasons). Of the remaining 296 (from 290 general practices), 15 were lost to follow-up, seven stopped using OAT, and seven stopped self-monitoring; this gave a total of 267 (90.2%) who were still self-monitoring at 12 months (Figure 1). Of those who stopped self-monitoring, four did so due to lack of healthcare provider support, two were unable to use the monitor reliably, and one was for unknown reasons.

### Baseline characteristics

The baseline characteristics of the cohort are presented in Table 1. In total, 51.2% were new to self-monitoring. The median target INR was 2.5 (interquartile range [IQR] 2.5–3.2). This varied by condition as follows:

- atrial fibrillation: median INR target of 2.5 (IQR 2.5–2.5);
- mechanical heart valve: median INR target of 3.0 (IQR 3.0–3.5);
- thrombosis: median INR target of 2.5 (IQR 2.5–3.0); and
- antiphospholipid syndrome: median INR target of 3.5 (IQR 2.5–3.8).

**Table 1.** Baseline characteristics

**Baseline characteristic**	***n***	**Total cohort^a^**	***n***	**New to self-monitoring^b^**	***n* Ongoing self-monitoring^c^**		***P* -value^d^**
**Demographic**							
Age, median (IQR)	295	61.0 (50.0–68.0)	152	59.0 (49.0–66.0)	143	63.0 (52.0–69.0)	0.031
Male, *n* (%)	296	163 (55.1)	152	88 (57.9)	144	75 (52.1)	0.315
White ethnicity, n (%)	294	293 (99.7)	150	149 (99.3)	144	144 (100.0)	1.000

**Highest educational qualification attained**	294		152		143		0.582
No qualification, *n* (%)		16 (5.4)		10 (6.6)		6 (4.2)	
O-Level or GCSE, *n* (%)		22 (7.5)		10 (6.6)		12 (8.4)	
A-Level, *n* (%)		13 (4.4)		8 (5.3)		5 (3.5)	
Professional qualification, *n* (%)		91 (31.0)		42 (27.8)		49 (34.3)	
University degree, *n* (%)		152 (51.7)		81 (53.6)		71 (49.7)	

**Condition requiring anticoagulation**	296		152		144		0.403
Antiphospholipid syndrome, *n* (%)		25 (8.4)		13 (8.6)		12 (8.3)	
Atrial fibrillation, *n* (%)		68 (23.0)		40 (26.3)		28 (19.4)	
Mechanical heart valve, *n* (%)		97 (32.8)		44 (28.9)		53 (36.8)	
Thrombosis, *n* (%)		106 (35.8)		55 (36.2)		51 (35.4)	

**Oral anticoagulation history**							
Target INR, median (IQR)	296	2.5 (2.5–3.2)	152	2.5 (2.5–3.0)	144	3.0 (2.5–3.3)	0.088
Duration of OAT, months, median (IQR)	295	62.0 (14.0–137.0)	151	21.0 (7.0–74.0)	144	107.0 (56.3–162.5)	<0.001
Previous OAT complication, *n* (%)	295	101 (34.2)	152	48 (31.6)	143	53 (37.1)	0.321
Taking OAT other than warfarin, *n* (%)	296	10 (3.4)	152	4 (2.6)	144	6 (4.2)	0.469

**Characteristics of self-monitoring**							
Duration, months, median (IQR)	295	2.0 (1.0–65.0)	151	1.0 (0.0–1.0)	144	65.0 (8.8–94.5)	<0.001
Received in-person self-monitoring training, *n* (%)	296	136 (45.9)	152	69 (45.4)	144	67 (46.5)	0.845
Duration of in-person training for those that received it in minutes, median (IQR)	129	35.0 (20.0–60.0)	66	30.0 (20.0–60.0)	63	45.0 (20.0–80.0)	0.010

**Other medications**							
Total medications, median (IQR)	296	3.0 (1.0–5.0)	152	3.0 (1.0–5.0)	144	3.0 (1.0–6.0)	0.213
Polypharmacy (total >3), *n* (%)	296	129 (43.6)	152	62 (40.8)	144	67 (46.5)	0.320
Taking medication that affects anticoagulation, *n* (%)	296	167 (56.4)	152	83 (54.6)	144	84 (58.3)	0.580

**Oral anticoagulation dose management**	296		152		144		<0.001
Self-testing, *n* (%)		133 (44.9)		79 (52.0)		54 (37.5)	
Mixed, *n* (%)		47 (15.9)		34 (22.4)		13 (9.0)	
Self-managing, *n* (%)		116 (39.2)		39 (25.7)		77 (53.5)	

a n = 296.

b n = 152.

c n = 144.

d P-values calculated using χ^2^ for categorical variables and binary logistic regression for continuous variables. GCSE = General Certificate of Secondary Education. IQR = interquartile range. OAT = oral anticoagulation therapy.

Just under half of participants self-monitored but received assistance with dose adjustment (133/296, 44.9%), while others self-managed and adjusted their own medication (116/296, 39.2%). A smaller number (47/296, 15.9%) did a mixture of both. Those new to self-monitoring were significantly less likely to be self-managing (25.7%, *P*<0.001) than those who had been self-monitoring for a longer period of time (53.5%).

Table 2 details participants’ psychosocial characteristics. More than half had problems in one or more dimensions of the EQ-5D (153/292, 52.4%), with the largest percentage being for those experiencing pain (122/296, 41.2%). Those who had been self-monitoring for longer had higher self-efficacy scores (*P* = 0.003), higher social pressure scores (*P* = 0.030), felt more in control of their illness (*P* = 0.014), and thought that their treatment controlled their illness more (*P* = 0.020). According to the NICE depression screening tool just over one-quarter (26.9%) of the participants were depressed, however, according to their HADS scores they were no more depressed and were slightly less anxious than the general population (Appendix 1).

**Table 2.** Psychosocial factors

**Psychosocial factors**	***n***	**Total cohort^a^**	***n***	**New to self-monitoring^b^**	***n***	**Ongoing self-monitoring^c^**	***P* -value^d^**
**EQ-5D**							
Total score, median (IQR)	292	6.0 (5.0–8.0)	150	6.0 (5.0–8.0)	142	5.0 (5.0–7.3)	0.401
Problem in any dimension, *n* (%)	292	153 (52.4)	150	87 (58.0)	142	66 (46.5)	0.049
Mobility problem, *n* (%)	296	88 (29.7)	152	47 (30.9)	144	41 (28.5)	0.645
Self-care problem, *n* (%)	296	31 (10.5)	152	15 (9.9)	144	16 (11.1)	0.727
Problem with usual activities, *n* (%)	295	92 (31.2)	152	53 (34.9)	143	39 (27.3)	0.160
Pain, *n* (%)	296	122 (41.2)	152	67 (44.1)	144	55 (38.2)	0.304
Anxiety problem, *n* (%)	293	69 (23.5)	150	41 (27.3)	143	28 (19.6)	0.119
Visual scale, median (IQR)	292	80.0 (65.0–90.0)	150	78 (60.0–90.0)	142	80.0 (69.8–90.0)	0.156

**Depression and anxiety**							
Depressed NICE screening tool, *n* (%)	294	79 (26.9)	150	47 (31.3)	144	32 (22.2)	0.079
Depressed assessed via HADS, *n* (%)	295	46 (15.6)	152	26 (17.1)	143	20 (14.0)	0.461
Anxious assessed via HADS, *n* (%)	295	70 (23.7)	152	34 (22.4)	143	36 (25.2)	0.571
STAI trait anxiety score, median (IQR)	295	33.0 (28.0–44.0)	151	32.0 (27.0–44.0)	144	33.0 (29.0–44.0)	0.878

**Self-determination theory**							
Autonomy score, median (IQR)	295	4.8 (3.8–5.5)	151	4.8 (4.0–5.5)	144	4.8 (3.8–5.5)	0.415
Control score, median (IQR)	295	1.0 (1.0–2.5)	151	1.3 (1.0–2.3)	144	1.0 (1.0–2.5)	0.680
Relative autonomy index, median (IQR)	295	3.0 (1.8–4.0)	151	3.0 (2.0–4.3)	144	2.9 (1.5–3.8)	0.307
Self-efficacy maximum marks, *n* (%)	296	250 (84.5)	152	119 (78.3)	144	131 (91.0)	0.003
Health care climate score, median (IQR)	294	34.0 (24.0–41.0)	150	33.0 (20.0–41.0)	144	34.0 (27.0–42.0)	0.185

**Theory of planned behaviour**							
Attitude score, median (IQR)	296	6.0 (5.5–7.0)	152	6.0 (5.5–7.0)	144	6.0 (5.5–7.0)	0.309
Social pressure score, median (IQR)	296	5.0 (4.0–7.0)	152	4.5 (4.0–6.4)	144	5.5 (4.0–7.0)	0.030
Control score, median (IQR)	296	7.0 (6.5–7.0)	152	7.0 (6.5–7.0)	144	7.0 (7.0–7.0)	0.099
Intention score, median (IQR)	296	7.0 (7.0–7.0)	152	7.0 (7.0–7.0)	144	7.0 (7.0–7.0)	0.526

**Illness perception**							
Consequences, median (IQR)	295	3.0 (2.0–7.0)	152	4.5 (2.0–7.0)	143	3.0 (1.0–6.0)	0.600
Timeline, median (IQR)	295	10.0 (10.0–10.0)	152	10.0 (10.0–10.0)	143	10.0 (10.0–10.0)	0.002
Personal control, median (IQR)	294	6.0 (3.0–8.0)	152	6.0 (3.0–8.0)	142	7.0 (4.0–8.0)	0.014
Treatment control, median (IQR)	295	9.0 (8.0–10.0)	152	9.0 (7.0–10.0)	143	9.0 (8.0–10.0)	0.020
Identity, median (IQR)	295	3.0 (1.0–6.0)	152	3.0 (1.0–6.0)	143	2.0 (1.0–6.0)	0.150
Concern, median (IQR)	295	5.0 (2.0–8.0)	152	6.0 (3.0–8.0)	143	4.0 (2.0–7.0)	0.008
Understanding, median (IQR)	295	9.0 (8.0–10.0)	152	9.0 (8.0–10.0)	143	10.0 (8.0–10.0)	0.719
Emotional response, median (IQR)	294	3.0 (1.0–6.0)	151	4.0 (1.0–7.0)	143	2.0 (0.0–5.0)	0.001

**Other**							
Requiring social assistance, *n* (%)	294	83 (28.2)	150	44 (29.3)	144	39 (27.1)	0.668
Conscientiousness, median (IQR)	282	41.0 (36.0–45.0)	143	42.0 (36.0–46.0)	139	41.0 (36.0–45.0)	0.878
Perfect medication adherence score, *n* (%)	288	176 (61.1)	149	94 (63.1)	139	82 (59.0)	0.856

a n = 296.

b n = 152.

c n = 144.

d P-values calculated using χ^2^ for categorical variables and binary logistic regression for continuous variables. HADS = Hospital Anxiety and Depression Scale. IQR = interquartile range. NICE = National Institute for Health and Care Excellence. STAI = State-Trait Anxiety Inventory.

### Training details

Nearly all participants (92.9%) made use of the information book and/or DVD that came with the monitor. However, only 45.9% of participants had received in-person training with a median duration of 35 minutes. Only six of 39 (15.4%) of those new to self-monitoring who were also self-managing had received any in-person training at baseline. Those new to self-monitoring who were self-testing or doing a mixture were more likely to receive training (59.5% and 47.1% respectively). The majority of this training took place in an outpatient hospital setting (62.5%), followed by primary care (36.8%); the remainder received training in both settings. Throughout the study period, 36/267 (13.5%) participants received additional training.

### Knowledge of oral anticoagulation

At 12 months, the participants had a high level of knowledge about OAT on the 20-item questionnaire (median score 18.0, IQR 16.0–19.0). There was no difference between those new to self-monitoring and those ongoing with self-monitoring. There was also no association between knowledge score and receiving in-person training (*P* = 0.658) or >80%TTR (*P* = 0.370). Areas where knowledge was lower were:

- diet and supplements;
- additional medication;
- testing frequency; and
- when to seek medical attention.

### Adverse events

Reports on adverse events were obtained from the GPs of 255 (86.1%) participants. Six (2.4%) participants experienced a serious adverse event — two deaths (one cause not reported, one from bowel cancer), two major bleeds, one thrombosis, and one transient ischaemic attack (TIA) — two experienced a minor bleed, and 18 experienced bleeds that were adjudicated to be less minor, as defined by the bleeding severity index. There were no significant differences in baseline characteristics between those with (*n* = 255) and without (*n* = 41) GP reports.

Twenty-eight participants self-reported adverse events, comprising serious adverse events (one TIA, three major bleeds), minor bleed (*n* = 4), and sub-minor bleed (*n* = 20). Of these, one serious adverse event (major bleed while participant was abroad), three minor bleeds, and 15 sub-minor bleeds were not reported by the participant’s GP. There were no significant differences between those new to, and those ongoing with, self-monitoring in terms of adverse events. There were also no differences between those who were self-managing and those who were self-testing.

### Frequency of testing

Participants reported a median of 10.9 days between tests (IQR 7.2–17.4). Those who had been self-monitoring for longer tested marginally more frequently (median 11.4 versus 10.5 days). Frequency of testing varied by condition:

- antiphospholipid syndrome: tested most frequently, median days between tests 8.0 (IQR 4.4–10.8);
- mechanical heart valve: median days between tests 10.8 (IQR 7.0–14.6);
- thrombosis: median days between tests 12.6 (IQR 7.5–18.8); and
- atrial fibrillation: median days between tests 13.2 (IQR 7.9–18.4).

Those who were only self-testing had the longest time between tests (median days between tests 14.7, IQR 9.7–21.1) and those self-managing the shortest (median days between tests 8.3, IQR 6.0–13.5); those undertaking a mixture were situated in between (median days between tests 9.8, IQR 5.8–15.9).

### Quality assurance

Nearly two-thirds (171/267, 64.0%) had performed an external calibration check of their device during the 12-month period. Of these, 50.8% compared their monitor with another monitor, 43.2% compared it with a venous sample, and 3.5% compared it with both; 2.3% were unknown. The majority (*n* = 117, 68.4%) had been advised to perform quality-assurance checks by their healthcare provider; the remainder used their initiative and coincided a self-monitoring test with a clinic monitoring appointment. The number of quality-assurance checks performed during the 12 months ranged from 1 to 24 (median 2, IQR 1–4).

### Time in therapeutic range

INR data was received from 273 participants (92.2% of the 296 who started self-monitoring) and was analysed for 269 (90.9%). Median %TTR was 78.5% (IQR 64.9–88.5). Table 3 shows that the older age groups had the highest %TTR. There was little difference in %TTR between those new to self-monitoring (median 76.1%, IQR 65.0–87.0) and those continuing to self-monitor (median 80.1%, IQR 64.8–91.4).

**Table 3.** Percentage of time spent in therapeutic range

**Age, years**	***n***	**% TTR, median (IQR)**	**Range**
<39	23	75.0 (64.8–83.1)	15.3–100.0
40–49	38	71.9 (58.9–86.0)	24.9–100.0
50–59	56	76.6 (62.4–85.8)	26.1–98.4
60–69	100	78.9 (62.1–90.4)	28.4–100.0
70–79	45	85.0 (73.4–90.6)	23.8–100.0
≥80	7	90.7 (77.3–95.9)	54.9–100.0
All ages	269	78.5 (64.9–88.5)	15.3–100.0

IQR = interquartile range. TTR = time in therapeutic range.

A total of 61 participants reported differing therapeutic ranges over the year. The breadth of INR target ranges started from as low as 0.4; the majority of cases (*n* = 243/296, 82.1%) had a range of 1 and the broadest range was 2. At 12 months, GPs were asked for their patient’s target range. These were compared with the target ranges reported by the participants at either the 12-month telephone interview or the last completed interview; for 80 participants (80/255, 31.4%) target ranges did not match. In 27 instances the participant reported a narrower range and in 25 the GP reported a narrower range; in the remaining 28 cases the breadth was the same but the actual range was different.

The participants’ 12-month mean %TTR (mean 75.3%, standard deviation 16.9) was higher than that achieved by the intervention groups in five^21^,^23^,^25^–^27^ of the seven 21–27 published RCTs.

### Successful self-monitoring

The majority of participants who commenced self-monitoring were still doing so 12 months later (267/296, 90.2%). In addition, the majority of participants did not experience serious adverse events over the 12 months (6/296, 2.0%) either GP or self-reported. Therefore, the main measure of successful self-monitoring was taken as the percentage of time in therapeutic range (TTR). Those who achieved >80% (n=128/269, 47.5%) of their time during follow-up in therapeutic range were defined as successful. Table 4 shows the logistic regressions, including the fixed variables and the baseline characteristics, that significantly differed at univariate level between those achieving ≥80% TTR and those with >80% TTR over the 12-month study period.

**Table 4.** Logistic regression predicting those with >80% time in therapeutic range

	**Odds ratio**	**95% CI**	***P*-value**
**All participants (*n* = 269)**			

Age, years^a^	1.024	1.000 to 1.048	0.052

Male^a^	1.320	0.753 to 2.312	0.332

**Condition requiring oral anticoagulation therapy^a^**			
Antiphospholipid syndrome	1.000	–	
Atrial fibrillation	5.231	1.249 to 21.904	0.024
Mechanical heart valve	3.685	0.933 to 14.555	0.063
Thrombosis	4.684	1.209 to 18.149	0.025

Duration of self-monitoring, months^a^	1.004	0.998 to 1.010	0.225

**Procedure during follow-up^a^**			
Major	1.000	–	
None	2.181	0.907 to 5.242	0.081
Minor	0.885	0.270 to 2.889	0.840

Previous OAT complication^a^	0.630	0.352 to 1.126	0.119

EQ-5D visual scale	1.017	1.001 to 1.034	0.032

**Participants with antiphospholipid syndrome excluded (*n* = 245)**			

Age, years^a^	1.025	1.003 to 1.049	0.027

Male^a^	1.303	0.736 to 2.306	0.364

Duration of self-monitoring, months^a^	1.003	0.997 to 1.010	0.295

**Procedure during follow-up^a^**			
Major	1.000	–	
None	2.023	0.836 to 4.893	0.118
Minor	0.734	0.221 to 2.443	0.614

Previous OAT complication^a^	0.752	0.412 to 1.374	0.354

EQ-5D visual scale	1.019	1.003 to 1.036	0.020

Target INR	0.480	0.246 to 0.939	0.032

a Variables the model was required to include. INR = international normalised ratio. OAT = oral anticoagulation therapy.

Results show that an increased EQ-5D visual scale was significantly associated with >80% TTR (OR 1.017, 95% CI = 1.001 to 1.034) as well as the forced variables of age and condition requiring oral anticoagulation. As antiphospholipid syndrome causes increased variability in INR the analysis was repeated removing these participants (*n* = 24). This resulted in the target INR becoming significant rather than the condition requiring anticoagulation, with those conditions with the lowest target ranges resulting in higher %TTR. Again age and a higher EQ-5D visual score were associated with higher %TTR.

## DISCUSSION

### Summary

These results demonstrate that patients can successfully self-monitor their INR outside trial settings. Successful self-monitoring was defined as having >80% TTR; nearly one-half of participants met this criterion. Increased age, OAT for atrial fibrillation or thrombosis, and having a higher score on the EQ-5D visual scale were all associated with success. When the participants with antiphospholipid syndrome were removed from the model only increased age and EQ-5D visual scale and lower target INR range were associated with success.

Of concern is that less than one-half the participants had received in-person training (46%) and, when it was present, this lasted, on average, for <1 hour. Instead, individuals appeared to rely solely on the training information that came with their device when it was purchased. Anticoagulation services varied in the support offered to patients wishing to self-monitor. Some had established systems, including training schemes, in place. In other areas, particularly those in which local GP surgeries were commissioned to provide anticoagulation services for their patients, approval to self-monitor was done on a more ad hoc basis without any dedicated schemes set up. Moreover, only 15% of those new to self-monitoring who were adjusting their own dosage of anticoagulation therapy had received in-person training when they commenced self-monitoring.

This cohort comprised well-educated, professional people, who successfully managed their self-monitoring with relatively little training; they also had a high level of knowledge about oral anticoagulation which was not related to training. Participants were aware of quality assurance and nearly two-thirds had performed an external calibration check of their device over the 12 months. In addition, they were similar to the general population in terms of anxiety and depression. A small number of participants could not commence self-monitoring or dropped out due to lack of healthcare provider support.

### Strengths and limitations

To the authors’ knowledge this is the first prospective cohort study of people self-monitoring their INR outside of trial conditions; the modified study design allowed for comparisons between those new to self-monitoring and those who had been self-monitoring for some time.

A limitation of the study is that, due to the fact that self-monitoring INR devices are expensive (approximately £400 in the UK), the participants were those people who were well educated and able to afford the device. This is of concern as the results may, therefore, not be generalisable to the wider population and current provision may be widening inequalities in health care.

Another potential limitation is the fact that the study, in part, relied on participants’ self-reported INR results. However, the accuracy of these have been confirmed in a small sample in which the reported results were compared to those stored in the memory of their monitor.

### Comparison with existing literature

The majority of participants were still self-monitoring at 12 months, with a level of control higher than that achieved by the majority of participants in other RCTs.^21^–^27^ These results are consistent with the findings of a large retrospective study^28^ of patient self-testing, which showed a high level of control in males (76%TTR), females (71%TTR), and older patients (>74 years, >73% TTR). In addition, the low number of adverse events compares favourably with rates for those in the intervention arms of the RCTs (7.5%).^3^

### Implications for practice

This study reliably shows that participants can effectively self-monitor their own INR, but current provision is limited due to the initial costs of the device and the lack of healthcare provider support.

Even with little training, people on OAT can successfully self-monitor and self-manage their INR; however, the fact that one-third of the participants gave different target INR ranges from the ones provided by their GP at the end of this study suggests that closer monitoring by healthcare providers may be warranted.

## Appendix 1. Baseline self-rated anxiety and depression of the CASM cohort members compared to population data using the Hospital Anxiety and Depression Score^a^

**Table:**

	**Male**		**Female**	
	
	**CASM (*n* = 162)**	**Population^b^**	**CASM (*n* = 133)**	**Population^b^**
HADS anxiety score, median (IQR)	4.0 (2.0–7.0)	5.0 (3.0–8.0)	5.0 (3.0–8.0)	6.0 (4.0–9.0)
HADS depression score, median (IQR)	3.0 (1.0–5.0)	3.0 (1.0–6.0)	3.0 (1.0–6.0)	3.0 (1.0–6.0)

IQR = interquartile range.

a A score of 0–7 is regarded as being normal, ≥8 indicates the presence of anxiety or depression.

*Population data from: Crawford JR, Henry JD, Crombie C, Taylor EP. Normative data for the HADS from a large non-clinical sample.* Br J Clin Psychol *2001;* ***40(Pt 4):*** *429–434.*
